# Neck Rejuvenation With a New Infrared Emission

**DOI:** 10.5826/dpc.1202a44

**Published:** 2022-04-01

**Authors:** Olga Mastrangelo, Luigi Bennardo, Irene Fusco, Mario Sannino, Giovanni Cannarozzo, Steven Paul Nisticò

**Affiliations:** 1 University of Rome Tor Vergata, Rome, Italy; 2 Department of Health Sciences, Unit of Dermatology, Magna Graecia University, Catanzaro, Italy; 3 University of Florence, Florence, Italy

**Keywords:** ipl, facial aging, infrared emission, wrinkles

## Introduction

Human skin aging includes histological and biochemical changes. The therapeutic technology of non-invasive skin rejuvenation of Intense Pulsed Light (IPL) is called photorejuvenation, and the technique has been used widely in cosmetic dermatology to improve facial photoaging. IPL sources are multiwavelength lights that typically emit light in the 500 to 1200 nm range. At this level, the thermal impulse causes denaturation of collagen fibers with consequent formation of new ones [1]. Papers that elucidate how systems emitting light near-infrared (800–1200 nm) could produce, on human skin fibroblasts cell cultures, dermal changes in gene expression and extracellular matrix and contribute to photo-rejuvenation are already present in literature [2].

## Case Presentation

**Figure 1:**
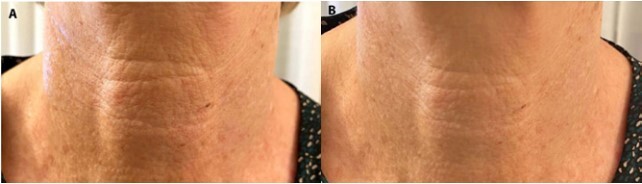
(A) Frontal view before treatment. (B) Frontal view after treatment

We present our preliminary experience with a 63-year-old female patient treated with a new pulsed infrared emission in the range 800–1200 nm (Luxea, Deka Mela Srl). Four sessions spaced 2 weeks apart were performed. The patient was recruited at Magna Graecia University of Catanzaro and signed informed consent. Local ethical committee approved the treatment protocol. The patient was photographed at the beginning and 3 months after the last treatment session (Figure 1).

The system is based on the pulsed emission of a wavelength range of 800–1200 nm over a 6.2 cm^2^ spot. The protocol used the following settings: power 30 W, handpiece moving in a linear slow motion creating an area about 5 × 5 cm. The handpiece is kept in a vertical position and in contact with the skin by applying light pressure and transparent water gel so that the entire surface of the irradiation area is always in contact with the skin. The protocol provides a progressive skin temperature rise of the epidermis up to 40–42 °C, persisting, as long as conditions allow, for a few minutes on the treatment area. The patient achieved an improvement in skin texture. A better skin tone and wrinkle reduction were observed in the neck area.

Patient in the immediate post-treatment experienced an improvement in brightness and porosity of the skin. The immediate effects are visibly enhanced 2 months after the first treatment session. The response of the dermal tissue is noticeable within 5 minutes. The endpoint was considered light erythema associated with the sensation of heat reported by the patient that disappeared in 30 minutes.

## Conclusions

Light devices emitting near-infrared are highly effective for skin rejuvenation. These treatments are associated with minimal patient discomfort and are well tolerated. In this context, patients require increasingly effective treatments associated with minimal pain and with the lowest possible risk of side effects. The new pulsed infrared emission in the range 800–1200 nm could be an excellent non-invasive system. Furthermore, this emission mode is convenient in the neck area where the alternatives are few and still invasive. The strength of this treatment is the absence of downtime and side effects. These results promise a rapid spread of this technology and are the starting point for combined treatments to treat more complex diseases that require integrated approaches.
